# 2-[({[Bis(pyridin-2-yl)methylidene]hydrazinecarbonyl}hydrazinylidene)(pyridin-2-yl)methyl]pyridinium tetra­fluoro­borate

**DOI:** 10.1107/S1600536811025359

**Published:** 2011-07-02

**Authors:** Jie Zhang

**Affiliations:** a School of Environment Science and Spatial Informatics, China University of Mining and Technology, Xuzhou 221116, Jiangsu Province, People’s Republic of China

## Abstract

In the title compound, C_23_H_19_N_8_O^+^·BF_4_^−^, one pyridine N atom is protonated. Two intra­molecular N—H⋯N hydrogen bonds occur. In the crystal, inter­molecular N—H⋯N hydrogen bond links neighboring C_23_H_19_N_8_O^+^ units into cyclic supra­molecular dimers while C—H⋯O hydrogen bonds link the C_23_H_19_N_8_O^+^ units into a two-dimensional supra­molecular network structure.

## Related literature

For the synthesis and crystal structure of the precursor ligand, 1,3-bis­(bis­(2-pyrid­yl)methyl­ene)amino)­urea, see: Manoj *et al.* (2005 ▶). For a tetra­nuclear iron(II) complex based on a deriv­ative of the title compound, see: Wu *et al.* (2009 ▶).
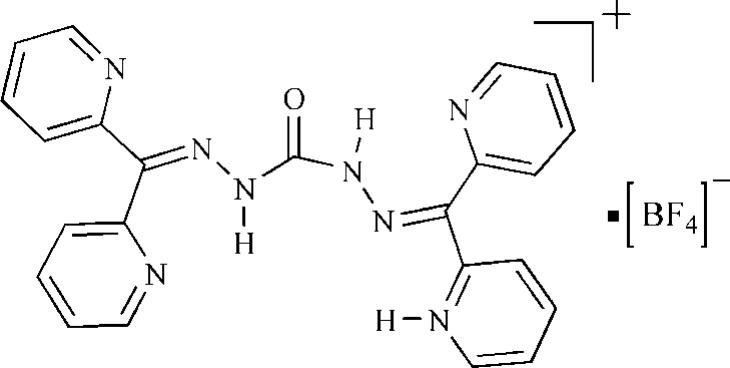


## Experimental

### 

#### Crystal data

C_23_H_19_N_8_O^+^·BF_4_^−^
*M* *_r_* = 510.27
Triclinic, 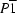

*a* = 7.9187 (16) Å
*b* = 10.626 (2) Å
*c* = 13.623 (3) Å
α = 90.03 (3)°
β = 91.50 (3)°
γ = 97.85 (3)°
*V* = 1135.1 (4) Å^3^
*Z* = 2
Mo *K*α radiation
μ = 0.12 mm^−1^
*T* = 123 K
0.22 × 0.19 × 0.16 mm

#### Data collection

Bruker APEXII CCD area-detector diffractometer
Absorption correction: multi-scan (*SADABS*; Sheldrick, 2003 ▶) *T*_min_ = 0.974, *T*_max_ = 0.981
18299 measured reflections
5175 independent reflections
4025 reflections with *I* > 2σ(*I*)
*R*_int_ = 0.032

#### Refinement

*R*[*F* ^2^ > 2σ(*F* ^2^)] = 0.048
*wR*(*F* ^2^) = 0.151
*S* = 0.98
5175 reflections
346 parameters
H atoms treated by a mixture of independent and constrained refinement
Δρ_max_ = 0.68 e Å^−3^
Δρ_min_ = −0.49 e Å^−3^

### 

Data collection: *APEX2* (Bruker, 2004 ▶); cell refinement: *SAINT-Plus* (Bruker, 2001 ▶); data reduction: *SAINT-Plus*; program(s) used to solve structure: *SHELXS97* (Sheldrick, 2008 ▶); program(s) used to refine structure: *SHELXL97* (Sheldrick, 2008 ▶); molecular graphics: *XP* in *SHELXTL* (Sheldrick, 2008 ▶); software used to prepare material for publication: *SHELXL97*.

## Supplementary Material

Crystal structure: contains datablock(s) global, I. DOI: 10.1107/S1600536811025359/ez2251sup1.cif

Structure factors: contains datablock(s) I. DOI: 10.1107/S1600536811025359/ez2251Isup2.hkl

Supplementary material file. DOI: 10.1107/S1600536811025359/ez2251Isup3.cml

Additional supplementary materials:  crystallographic information; 3D view; checkCIF report

## Figures and Tables

**Table 1:** Hydrogen-bond geometry (Å, °)

*D*—H⋯*A*	*D*—H	H⋯*A*	*D*⋯*A*	*D*—H⋯*A*
N1—H1⋯N7^i^	0.90 (2)	1.92 (2)	2.792 (2)	163.5 (15)
N3—H30⋯N8	0.88 (2)	1.99 (2)	2.658 (2)	132.1 (19)
N4—H40⋯N6	0.83 (2)	2.02 (2)	2.640 (2)	132 (2)
C2—H2⋯O1^ii^	0.93	2.41	3.083 (2)	129 (1)
C18—H18⋯O1^iii^	0.93	2.46	3.355 (2)	162 (1)

Symmetry codes: (i) 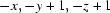; (ii) 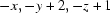; (iii) 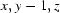.

**Fig. 1.**
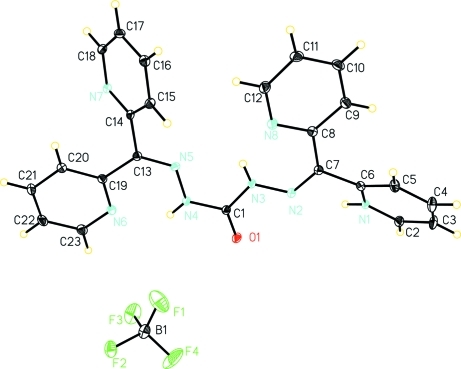
A view of (I) with the unique atom-labelling scheme. Displacement ellipsoids are drawn at the 30% probability level.

**Fig. 2.**
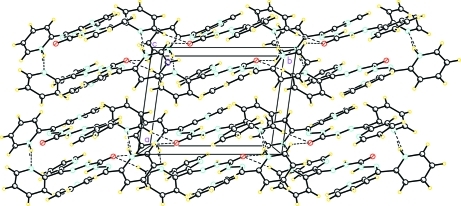
A view of part of the crystal structure of (I) along the c axis, showing two-dimensional supramolecular structure through formed by N—H···N and C—H···O hydrogen bonds.

## supplementary crystallographic information

### Comment

Recently, the ligand 1,3-bis(bis(2-pyridyl)methylene)amino)urea and its derivatives have been employed to assembly clusters with novel topological structures and interesting magnetic and photomagnetic properties (Wu *et al*., 2009). In our attempt to synthesize a tetranuclear square iron cluster based on the ligand 1,3-bis(bis(2-pyridyl)methylene)amino)urea and Fe^II^(BF_4_)_2_.4H_2_O, the title compound was obtained unexpectedly. Herein, we report the crystal structure of 1,3-bis(bis(2-pyridyl)methylene)amino)urea tetrafluoroborate (I).

The geometry and labeling scheme for the crystal structure of the title complex are depicted in Figure 1. The molecular structure comprises a [C_23_H_19_N_8_O]^+^ cation and a charge balancing anion [BF_4_]^-^. In the molecular structure, one pyridine nitrogen was protonated by one hydrogen. The cation adopts an *EE* configuration about the hydrazine bonds which is similar to its precursor 1,3-bis(bis(2-pyridyl)methylene)amino)urea (Manoj *et al.*, 2005).

The C—O bond length is 1.2157 (18) Å. The C—N_hydrazine_ double bond lengths are 1.297 (2) and 1.301 (2) Å, respectively. There are two intramolecular N—H···N hydrogen bonds. A relatively strong intermolecular N—H···N hydrogen bond links two neighboring [C_23_H_19_N_8_O]^+^ units into a cyclic supramolecular dimer. In addition, C—H···O hydrogen bonds link the [C_23_H_19_N_8_O]^+^ units into a two-dimensional supramolecular network structure as shown in Figure 2.

### Experimental

The title complex was prepared as following: a methanol solution (5 ml) of [Fe^II^(BF_4_)_2_].4H_2_O (60 mg, 0.2 mmol) was added slowly to a MeOH suspension (20 ml) containing the ligand 1,3-bis(bis(2-pyridyl)methylene)amino)urea (84 mg, 0.2 mmol). After stirring 30 min, the mixture was then carefully filtered and the resulting solution was kept at room temperature for about two days, producing colorless block-shape crystals of (I) with high yield (*ca* 60%).

### Refinement

The coordinates of the three H atoms bound to three nitrogen atoms were found from difference Fourier maps and refined freely. H atoms bound to C atoms were placed using the HFIX commands in *SHELXL-97*, with C—H distances of 0.93 Å. All H atoms were allowed for as riding atoms with *U*_iso_(H) = 1.2*U*_eq_(C).

### Crystal data

**Table:**

C_23_H_19_N_8_O^+^·BF_4_^−^	*Z* = 2
*M**_r_* = 510.27	*F*(000) = 524
Triclinic, *P*1	*D*_x_ = 1.493 Mg m^−^^3^
Hall symbol: -P 1	Mo *K*α radiation, λ = 0.71073 Å
*a* = 7.9187 (16) Å	Cell parameters from 3505 reflections
*b* = 10.626 (2) Å	θ = 2.3–26.7°
*c* = 13.623 (3) Å	µ = 0.12 mm^−^^1^
α = 90.03 (3)°	*T* = 123 K
β = 91.50 (3)°	Block, colorless
γ = 97.85 (3)°	0.22 × 0.19 × 0.16 mm
*V* = 1135.1 (4) Å^3^	

### Data collection

**Table:**

Bruker APEXII CCD area-detector diffractometer	5175 independent reflections
Radiation source: fine-focus sealed tube	4025 reflections with *I* > 2σ(*I*)
graphite	*R*_int_ = 0.032
φ and ω scans	θ_max_ = 27.5°, θ_min_ = 3.0°
Absorption correction: multi-scan (*SADABS*; Sheldrick, 2003)	*h* = −10→10
*T*_min_ = 0.974, *T*_max_ = 0.981	*k* = −13→13
18299 measured reflections	*l* = −17→17

### Refinement

**Table:**

Refinement on *F*^2^	Primary atom site location: structure-invariant direct methods
Least-squares matrix: full	Secondary atom site location: difference Fourier map
*R*[*F*^2^ > 2σ(*F*^2^)] = 0.048	Hydrogen site location: inferred from neighbouring sites
*wR*(*F*^2^) = 0.151	H atoms treated by a mixture of independent and constrained refinement
*S* = 0.98	*w* = 1/[σ^2^(*F*_o_^2^) + (0.1033*P*)^2^ + 0.3032*P*] where *P* = (*F*_o_^2^ + 2*F*_c_^2^)/3
5175 reflections	(Δ/σ)_max_ < 0.001
346 parameters	Δρ_max_ = 0.68 e Å^−^^3^
0 restraints	Δρ_min_ = −0.49 e Å^−^^3^

### Special details

**Table:**

Geometry. All e.s.d.'s (except the e.s.d. in the dihedral angle between two l.s. planes) are estimated using the full covariance matrix. The cell e.s.d.'s are taken into account individually in the estimation of e.s.d.'s in distances, angles and torsion angles; correlations between e.s.d.'s in cell parameters are only used when they are defined by crystal symmetry. An approximate (isotropic) treatment of cell e.s.d.'s is used for estimating e.s.d.'s involving l.s. planes.
Refinement. Refinement of *F*^2^ against ALL reflections. The weighted *R*-factor *wR* and goodness of fit *S* are based on *F*^2^, conventional *R*-factors *R* are based on *F*, with *F* set to zero for negative *F*^2^. The threshold expression of *F*^2^ > σ(*F*^2^) is used only for calculating *R*-factors(gt) *etc*. and is not relevant to the choice of reflections for refinement. *R*-factors based on *F*^2^ are statistically about twice as large as those based on *F*, and *R*- factors based on ALL data will be even larger.

### Fractional atomic coordinates and isotropic or equivalent isotropic displacement parameters (Å^2^)

**Table:**

	*x*	*y*	*z*	*U*_iso_*/*U*_eq_	
H1	−0.020 (3)	0.896 (2)	0.6632 (16)	0.033 (6)*	
H30	0.224 (3)	0.592 (2)	0.5650 (17)	0.041 (6)*	
H40	0.141 (3)	0.567 (2)	0.3285 (17)	0.038 (6)*	
O1	0.07813 (16)	0.75955 (10)	0.40804 (8)	0.0215 (3)	
N1	0.05189 (17)	0.93425 (12)	0.70991 (9)	0.0151 (3)	
N2	0.16150 (16)	0.75693 (12)	0.60077 (9)	0.0149 (3)	
N3	0.17522 (17)	0.65646 (12)	0.54249 (9)	0.0147 (3)	
N4	0.16693 (18)	0.56751 (12)	0.38758 (10)	0.0164 (3)	
N6	0.15116 (17)	0.43582 (12)	0.22247 (9)	0.0178 (3)	
N7	0.20613 (16)	0.14392 (12)	0.42932 (9)	0.0154 (3)	
N8	0.32921 (18)	0.55934 (13)	0.69471 (10)	0.0210 (3)	
N5	0.21894 (16)	0.46491 (12)	0.43220 (9)	0.0147 (3)	
C13	0.24217 (19)	0.36556 (14)	0.38154 (11)	0.0143 (3)	
C8	0.27284 (19)	0.65124 (14)	0.74875 (11)	0.0163 (3)	
C19	0.22420 (19)	0.34758 (14)	0.27322 (11)	0.0152 (3)	
C14	0.29165 (19)	0.26067 (14)	0.44455 (11)	0.0142 (3)	
C20	0.2835 (2)	0.24569 (15)	0.22615 (12)	0.0193 (3)	
H20	0.3378	0.1878	0.2621	0.023*	
C15	0.4159 (2)	0.28667 (15)	0.51871 (11)	0.0175 (3)	
H15	0.4729	0.3685	0.5276	0.021*	
C5	0.2903 (2)	0.93506 (16)	0.81632 (12)	0.0219 (4)	
H5	0.3817	0.8971	0.8408	0.026*	
C1	0.13426 (19)	0.66862 (14)	0.44403 (11)	0.0152 (3)	
C16	0.4535 (2)	0.18811 (15)	0.57937 (11)	0.0185 (3)	
H16	0.5366	0.2026	0.6291	0.022*	
C6	0.1830 (2)	0.87492 (14)	0.74368 (11)	0.0158 (3)	
C17	0.3646 (2)	0.06836 (15)	0.56384 (11)	0.0183 (3)	
H17	0.3867	0.0008	0.6033	0.022*	
C9	0.2753 (2)	0.64769 (16)	0.85125 (12)	0.0207 (3)	
H9	0.2326	0.7102	0.8871	0.025*	
C18	0.2421 (2)	0.05004 (14)	0.48887 (11)	0.0174 (3)	
H18	0.1821	−0.0308	0.4794	0.021*	
C7	0.20552 (19)	0.75424 (14)	0.69332 (11)	0.0151 (3)	
C12	0.3918 (2)	0.46578 (17)	0.74277 (13)	0.0261 (4)	
H12	0.4309	0.4025	0.7059	0.031*	
C2	0.0211 (2)	1.04672 (15)	0.74398 (12)	0.0208 (3)	
H2	−0.0712	1.0831	0.7189	0.025*	
C23	0.1328 (2)	0.42228 (16)	0.12492 (12)	0.0218 (4)	
H23	0.0845	0.4838	0.0896	0.026*	
C22	0.1824 (2)	0.32125 (17)	0.07416 (12)	0.0245 (4)	
H22	0.1634	0.3135	0.0066	0.029*	
C10	0.3423 (2)	0.54963 (16)	0.89862 (12)	0.0237 (4)	
H10	0.3467	0.5465	0.9669	0.028*	
C11	0.4023 (2)	0.45699 (16)	0.84425 (13)	0.0254 (4)	
H11	0.4485	0.3905	0.8745	0.030*	
C21	0.2605 (2)	0.23194 (16)	0.12549 (12)	0.0239 (4)	
H21	0.2970	0.1639	0.0930	0.029*	
C3	0.1261 (2)	1.10889 (17)	0.81633 (13)	0.0276 (4)	
H3	0.1061	1.1874	0.8401	0.033*	
C4	0.2611 (2)	1.05221 (18)	0.85257 (13)	0.0284 (4)	
H4	0.3330	1.0925	0.9015	0.034*	
B1	0.2959 (3)	0.8076 (2)	0.10945 (17)	0.0306 (5)	
F1	0.3407 (2)	0.7618 (2)	0.20065 (11)	0.0788 (5)	
F2	0.42071 (15)	0.78336 (12)	0.04479 (8)	0.0381 (3)	
F3	0.14286 (17)	0.73994 (14)	0.07990 (12)	0.0618 (4)	
F4	0.2908 (2)	0.93300 (14)	0.11734 (17)	0.0898 (7)	

### Atomic displacement parameters (Å^2^)

**Table:**

	*U*^11^	*U*^22^	*U*^33^	*U*^12^	*U*^13^	*U*^23^
O1	0.0329 (7)	0.0149 (6)	0.0180 (6)	0.0091 (5)	−0.0041 (5)	−0.0001 (4)
N1	0.0181 (7)	0.0144 (6)	0.0127 (6)	0.0024 (5)	−0.0015 (5)	−0.0014 (5)
N2	0.0157 (6)	0.0142 (6)	0.0147 (6)	0.0021 (5)	0.0002 (5)	−0.0019 (5)
N3	0.0194 (7)	0.0118 (6)	0.0135 (6)	0.0049 (5)	−0.0013 (5)	−0.0010 (5)
N4	0.0245 (7)	0.0134 (6)	0.0120 (6)	0.0052 (5)	−0.0028 (5)	−0.0002 (5)
N6	0.0194 (7)	0.0182 (7)	0.0157 (6)	0.0022 (5)	−0.0008 (5)	−0.0007 (5)
N7	0.0172 (6)	0.0135 (6)	0.0157 (6)	0.0031 (5)	0.0009 (5)	−0.0016 (5)
N8	0.0269 (8)	0.0194 (7)	0.0181 (7)	0.0082 (6)	−0.0022 (6)	−0.0005 (5)
N5	0.0164 (6)	0.0116 (6)	0.0160 (6)	0.0017 (5)	−0.0014 (5)	0.0013 (5)
C13	0.0139 (7)	0.0139 (7)	0.0147 (7)	0.0008 (6)	−0.0005 (6)	−0.0006 (6)
C8	0.0141 (7)	0.0181 (8)	0.0165 (7)	0.0019 (6)	−0.0017 (6)	0.0001 (6)
C19	0.0150 (7)	0.0151 (7)	0.0147 (7)	−0.0008 (6)	0.0006 (6)	−0.0001 (6)
C14	0.0158 (7)	0.0130 (7)	0.0145 (7)	0.0037 (6)	0.0028 (6)	−0.0017 (5)
C20	0.0224 (8)	0.0177 (8)	0.0180 (8)	0.0028 (6)	0.0036 (6)	0.0002 (6)
C15	0.0176 (8)	0.0151 (7)	0.0194 (8)	0.0012 (6)	−0.0011 (6)	−0.0011 (6)
C5	0.0204 (8)	0.0261 (9)	0.0191 (8)	0.0040 (7)	−0.0034 (6)	−0.0050 (6)
C1	0.0165 (7)	0.0138 (7)	0.0150 (7)	0.0015 (6)	−0.0007 (6)	−0.0008 (6)
C16	0.0180 (8)	0.0226 (8)	0.0158 (7)	0.0058 (6)	−0.0024 (6)	−0.0003 (6)
C6	0.0167 (7)	0.0172 (7)	0.0135 (7)	0.0025 (6)	0.0015 (6)	−0.0004 (6)
C17	0.0205 (8)	0.0181 (8)	0.0175 (7)	0.0064 (6)	0.0025 (6)	0.0050 (6)
C9	0.0222 (8)	0.0231 (8)	0.0172 (8)	0.0049 (7)	0.0000 (6)	0.0001 (6)
C18	0.0199 (8)	0.0130 (7)	0.0196 (8)	0.0028 (6)	0.0027 (6)	−0.0007 (6)
C7	0.0139 (7)	0.0157 (7)	0.0158 (7)	0.0020 (6)	0.0008 (6)	−0.0005 (6)
C12	0.0361 (10)	0.0214 (8)	0.0229 (9)	0.0128 (7)	−0.0034 (7)	−0.0009 (7)
C2	0.0259 (9)	0.0180 (8)	0.0196 (8)	0.0070 (6)	0.0004 (7)	−0.0017 (6)
C23	0.0246 (9)	0.0244 (8)	0.0159 (8)	0.0025 (7)	−0.0027 (6)	0.0009 (6)
C22	0.0321 (10)	0.0275 (9)	0.0125 (8)	−0.0011 (7)	0.0006 (7)	−0.0027 (6)
C10	0.0281 (9)	0.0266 (9)	0.0158 (8)	0.0020 (7)	−0.0031 (7)	0.0044 (6)
C11	0.0306 (9)	0.0218 (8)	0.0246 (9)	0.0080 (7)	−0.0058 (7)	0.0057 (7)
C21	0.0310 (9)	0.0212 (8)	0.0195 (8)	0.0024 (7)	0.0068 (7)	−0.0050 (6)
C3	0.0351 (10)	0.0214 (9)	0.0266 (9)	0.0056 (7)	−0.0011 (8)	−0.0122 (7)
C4	0.0287 (9)	0.0302 (9)	0.0252 (9)	0.0013 (7)	−0.0055 (7)	−0.0130 (7)
B1	0.0283 (11)	0.0281 (11)	0.0357 (12)	0.0049 (9)	−0.0021 (9)	−0.0079 (9)
F1	0.0849 (13)	0.1169 (15)	0.0361 (8)	0.0202 (11)	−0.0023 (8)	0.0033 (9)
F2	0.0329 (6)	0.0449 (7)	0.0360 (6)	0.0037 (5)	−0.0016 (5)	−0.0140 (5)
F3	0.0313 (7)	0.0607 (9)	0.0918 (12)	0.0014 (6)	−0.0033 (7)	−0.0231 (8)
F4	0.0708 (12)	0.0280 (8)	0.174 (2)	0.0124 (7)	0.0381 (12)	−0.0134 (10)

### Geometric parameters (Å, °)

**Table:**

O1—C1	1.2157 (18)	C5—C4	1.389 (2)
N1—C2	1.337 (2)	C5—H5	0.9300
N1—C6	1.357 (2)	C16—C17	1.381 (2)
N1—H1	0.90 (2)	C16—H16	0.9300
N2—C7	1.301 (2)	C6—C7	1.488 (2)
N2—N3	1.3481 (18)	C17—C18	1.385 (2)
N3—C1	1.3820 (19)	C17—H17	0.9300
N3—H30	0.89 (2)	C9—C10	1.385 (2)
N4—N5	1.3557 (17)	C9—H9	0.9300
N4—C1	1.378 (2)	C18—H18	0.9300
N4—H40	0.82 (2)	C12—C11	1.387 (2)
N6—C23	1.339 (2)	C12—H12	0.9300
N6—C19	1.349 (2)	C2—C3	1.380 (2)
N7—C18	1.3418 (19)	C2—H2	0.9300
N7—C14	1.343 (2)	C23—C22	1.383 (2)
N8—C12	1.335 (2)	C23—H23	0.9300
N8—C8	1.353 (2)	C22—C21	1.383 (2)
N5—C13	1.297 (2)	C22—H22	0.9300
C13—C19	1.489 (2)	C10—C11	1.374 (3)
C13—C14	1.495 (2)	C10—H10	0.9300
C8—C9	1.396 (2)	C11—H11	0.9300
C8—C7	1.480 (2)	C21—H21	0.9300
C19—C20	1.399 (2)	C3—C4	1.378 (3)
C14—C15	1.392 (2)	C3—H3	0.9300
C20—C21	1.384 (2)	C4—H4	0.9300
C20—H20	0.9300	B1—F4	1.342 (3)
C15—C16	1.393 (2)	B1—F3	1.373 (3)
C15—H15	0.9300	B1—F1	1.390 (3)
C5—C6	1.384 (2)	B1—F2	1.391 (3)
			
C2—N1—C6	123.20 (14)	C16—C17—H17	120.4
C2—N1—H1	117.6 (14)	C18—C17—H17	120.4
C6—N1—H1	119.2 (14)	C10—C9—C8	118.98 (15)
C7—N2—N3	120.02 (13)	C10—C9—H9	120.5
N2—N3—C1	116.77 (13)	C8—C9—H9	120.5
N2—N3—H30	120.6 (15)	N7—C18—C17	122.81 (14)
C1—N3—H30	121.9 (15)	N7—C18—H18	118.6
N5—N4—C1	119.28 (13)	C17—C18—H18	118.6
N5—N4—H40	121.7 (16)	N2—C7—C8	128.54 (14)
C1—N4—H40	118.4 (16)	N2—C7—C6	111.30 (13)
C23—N6—C19	118.19 (14)	C8—C7—C6	120.15 (13)
C18—N7—C14	118.10 (13)	N8—C12—C11	124.13 (16)
C12—N8—C8	117.68 (14)	N8—C12—H12	117.9
C13—N5—N4	120.72 (13)	C11—C12—H12	117.9
N5—C13—C19	127.73 (14)	N1—C2—C3	120.10 (16)
N5—C13—C14	112.55 (13)	N1—C2—H2	119.9
C19—C13—C14	119.72 (13)	C3—C2—H2	119.9
N8—C8—C9	121.70 (14)	N6—C23—C22	123.18 (16)
N8—C8—C7	116.38 (13)	N6—C23—H23	118.4
C9—C8—C7	121.90 (14)	C22—C23—H23	118.4
N6—C19—C20	121.65 (14)	C21—C22—C23	118.89 (15)
N6—C19—C13	116.70 (13)	C21—C22—H22	120.6
C20—C19—C13	121.63 (14)	C23—C22—H22	120.6
N7—C14—C15	122.42 (14)	C11—C10—C9	119.63 (15)
N7—C14—C13	117.28 (13)	C11—C10—H10	120.2
C15—C14—C13	120.22 (13)	C9—C10—H10	120.2
C21—C20—C19	119.31 (15)	C10—C11—C12	117.84 (15)
C21—C20—H20	120.3	C10—C11—H11	121.1
C19—C20—H20	120.3	C12—C11—H11	121.1
C14—C15—C16	118.96 (14)	C22—C21—C20	118.71 (15)
C14—C15—H15	120.5	C22—C21—H21	120.6
C16—C15—H15	120.5	C20—C21—H21	120.6
C6—C5—C4	119.88 (16)	C4—C3—C2	118.64 (16)
C6—C5—H5	120.1	C4—C3—H3	120.7
C4—C5—H5	120.1	C2—C3—H3	120.7
O1—C1—N4	121.74 (14)	C3—C4—C5	120.25 (16)
O1—C1—N3	124.77 (14)	C3—C4—H4	119.9
N4—C1—N3	113.48 (13)	C5—C4—H4	119.9
C17—C16—C15	118.44 (14)	F4—B1—F3	113.43 (19)
C17—C16—H16	120.8	F4—B1—F1	108.7 (2)
C15—C16—H16	120.8	F3—B1—F1	107.76 (19)
N1—C6—C5	117.93 (14)	F4—B1—F2	110.79 (19)
N1—C6—C7	116.20 (13)	F3—B1—F2	108.98 (17)
C5—C6—C7	125.70 (14)	F1—B1—F2	106.95 (17)
C16—C17—C18	119.25 (14)		

### Hydrogen-bond geometry (Å, °)

**Table:**

*D*—H···*A*	*D*—H	H···*A*	*D*···*A*	*D*—H···*A*
N1—H1···N7^i^	0.90 (2)	1.92 (2)	2.792 (2)	163.5 (15)
N3—H30···N8	0.88 (2)	1.99 (2)	2.658 (2)	132.1 (19)
N4—H40···N6	0.83 (2)	2.02 (2)	2.640 (2)	132 (2)
C2—H2···O1^ii^	0.93	2.41	3.083 (2)	129.(1)
C18—H18···O1^iii^	0.93	2.46	3.355 (2)	162.(1)

Symmetry codes: (i) −*x*, −*y*+1, −*z*+1;  (ii) −*x*, −*y*+2, −*z*+1; (iii) *x*, *y*−1, *z*.
